# Optimization of the Fermentation Process for a Mulberry Beverage Using Composite Microbial Strains and a Study on Its Physicochemical Properties

**DOI:** 10.3390/foods14244312

**Published:** 2025-12-15

**Authors:** Di Wang, Jingqiu Zhou, Wei Bu, Chong Ning

**Affiliations:** 1 College of Light Industry, Liaoning University, Shenyang 110036, China; 4032142754@smail.lnu.edu.cn (D.W.); zhoujqchn@126.com (J.Z.); 13994387375@163.com (W.B.); 2 College of Medicine and Biological Information Engineering, Northeastern University, Shenyang 110169, China

**Keywords:** composite microorganism fermentation, fermented beverage, physicochemical properties, volatile substances

## Abstract

Mulberries are rich in bioactive compounds and posse significant nutritional value. Fermentation can enhance the product’s flavor, while the synergistic effects of multiple strains can improve the quality of fruits and vegetables, resulting in a greater array of nutritional components. *Saccharomyces cerevisiae* and *Lactobacillus plantarum* were employed as fermentation strains to produce fermented mulberry beverages. Utilizing one-way ANOVA and an orthogonal experimental design, the optimal process conditions were established as follows: *Saccharomyces cerevisiae*–*Lactobacillus plantarum* ratio: 2:1 (*v*/*v*), inoculum amount: 1% (*v*/*v*), fermentation time: 60 h, and fermentation temperature: 30 °C. Under these conditions, the results showed a sugar content of 7.5 ± 0.2 °Brix, a pH of 4.12 ± 0.12, and a sensory evaluation score of 89 ± 1.34. The composite-strain fermented mulberry beverage exhibited a significantly elevated total anthocyanin content, along with enhanced SOD enzyme activity, DPPH radical scavenging capacity, and ABTS radical scavenging capacity. Volatile component analysis revealed that the composite-strain fermented mulberry beverage exhibited a more diverse range of aromatic compounds, with a total of 37 types, approximately 10 types more than both the mulberry juice and the single-strain fermented mulberry beverage. This study revealed the advantages of composite microbial fermentation, laying a foundation for the development of new-type high-quality fermented beverages.

## 1. Introduction

Mulberries (*Morus alba* L.) are rich in phenolic compounds, polysaccharides, and other nutrients, exhibiting significant antioxidant, anti-aging, and anti-inflammatory bioactivities. Their nutritional value has gained widespread recognition, making them increasingly popular [1,2]. They are extensively utilized in the food industry, with a focus on advancing the juice processing sector to support the healthful and sustainable expansion of the mulberry industry [3]. However, mulberries are a seasonal soft fruit prone to bruising and rapid spoilage, which leads to substantial post-harvest losses [4]. Therefore, efficient processing methods are needed to improve resource utilization and extend shelf life.

Therefore, fermentation, as an efficient bioconversion technology, enhances the nutritional properties, sensory qualities, and functional activity of fruits and vegetables while effectively extending product shelf life. Through microbial metabolism, various organic acids, free amino acids, aromatic compounds, and bioactive components are generated. This not only imparts unique flavors to products but also promotes the deep development and high-value utilization of fruit and vegetable resources [5,6]. These transformations result from the biochemical roles of different microbial groups involved in the fermentation process. Yeasts, particularly *Saccharomyces cerevisiae*, catabolize sugars into ethanol, carbon dioxide, and diverse aroma precursors, and convert amino acids into higher alcohols and esters [7]. However, it suffers from the drawback of producing a monotonous and convergent flavor profile. To generate more complex aromatic compounds, non-*Saccharomyces* are gaining increasing attention [8]. In contrast to yeast-driven pathways, lactic acid bacteria such as *Lactobacillus plantarum* contribute distinct metabolic functions that strongly influence both juice chemistry and bioactivity. They metabolize carbohydrates and amino acids into organic acids, thereby lowering pH, suppressing undesirable microorganisms, and shaping the characteristic acidic flavor of fermented fruit beverages [9]. In addition, *Lactobacillus* possess a broad repertoire of hydrolytic enzymes—including β-glucosidase and other polysaccharide—degrading enzymes—that depolymerize macromolecules and release bound phenolics and volatile precursors, thereby enhancing the bioavailability of phenolic compounds [10,11]. Previous studies found that *Lactobacillus* fermentation increased the contents of catechin and protocatechuic acid, which would be conducive to enhancing the antioxidant potential of kiwifruit juice [12]. Research by Tong et al. showed that fermenting strawberry juice with *L. plantarum* for 8–13 h significantly increased total acid content and volatile compound diversity while enhancing DPPH radical scavenging capacity [13]. Similarly, *L. plantarum* fermentation elevated lactic acid content, pH, and antioxidant activity in sweet lemon juice [14]. Because of its strong acid tolerance and extensive enzymatic capacity, *L. plantarum* has emerged as a promising probiotic strain for juice fermentation [15]. Nevertheless, single-strain fermentation often limits the comprehensive utilization of fruit substrates, making it difficult to simultaneously optimize both functional properties and flavor complexity [16].

Building on these characteristics, co-culture fermentation systems have garnered significant attention in recent years. By combining the metabolic activities of different microorganisms, co-cultures can drive substrate conversion more efficiently, increase metabolite diversity, and strengthen functional properties, thereby exhibiting clear metabolic complementarity [17]. Co-fermentation of *S. cerevisiae* and *L. plantarum* has been shown to improve antioxidant capacity and enhance free-radical-scavenging activity during apple juice fermentation [18]. Similarly, fermentation of pear juice with three lactic acid bacteria strains increased its antioxidant capacity and significantly enriched its flavor-compound profile [19]. In addition, fermenting fruit and vegetable juices with mixed cultures of lactic acid bacteria and yeast can generate a wider spectrum of amino acids, vitamins, digestive enzymes, and aroma-related metabolites, further contributing to improved nutritional and sensory qualities [6].

However, despite the growing interest in microbial co-fermentation, research involving mulberry juice is still limited. Most existing studies focus on single-strain fermentation, and little is known about how different microbial groups interact when fermenting mulberry substrates. In particular, although *S. cerevisiae* and *L. plantarum* have been individually studied in various fruit matrices, their combined effects on the physicochemical properties, bioactive compounds, antioxidant activity, and volatile profile of mulberry juice have not been comprehensively evaluated. Addressing this gap is important for developing mulberry-based fermented beverages with both improved functionality and enhanced flavor complexity.

Therefore, this study employed *L. plantarum* and *S. cerevisiae* to establish a co-cultured fermentation system. This combination was selected based on their complementary carbon utilization, metabolic pathways, and ecological niches: *L. plantarum* possesses strong organic acid synthesis and sugar degradation capabilities, improving substrate fermentability; while *S. cerevisiae* contributes to ethanol and ester synthesis, synergistically regulating the aroma profile [8]. Crucially, during co-fermentation, both strains jointly mitigate the oxidative degradation of polyphenols by enhancing the activity of endogenous antioxidant enzymes such as superoxide dismutase (SOD), thereby better preserving the antioxidant properties and nutritional integrity of mulberry juice [20]. This study aims to systematically evaluate the physicochemical characteristics, bioactive compounds, antioxidant activities, and volatile components of mulberry beverages fermented with single strains and with a co-culture system, in order to clarify the specific advantages of composite microbial fermentation. The results demonstrate that co-fermentation markedly enhances organic acid and anthocyanin levels, improves antioxidant capacity, and enriches the volatile flavor profile. These findings provide necessary scientific evidence supporting the development of high-quality fermented mulberry beverages with improved flavor and enhanced functionality.

## 2. Materials and Methods

### 2.1. Materials and Reagents

Fresh mulberries were obtained from Huludao, China. *Saccharomyces cerevisiae* (ATCC 2338) and *Lactobacillus plantarum* (ATCC 8014) were purchased from Beijing Biobank Biotechnology Co., Ltd., Beijing, China. All chemicals and reagents of analytical grade were purchased from Sinopharm Chemical Reagent Co., Ltd. (Shanghai, China) and Tianjin Yongda Chemical Reagent Co., Ltd. (Tianjin, China).

### 2.2. Preparation of Fermented Mulberry Beverage

Fresh mulberries with uniform color and ripeness were selected, washed thoroughly, and stored at −20 °C until further use. After thawing, the fruits were crushed to obtain the juice, which underwent enzymatic hydrolysis with pectinase (0.04%, *w*/*w*) at 50 °C for 150 min. Once cooled to room temperature, sucrose was added to adjust the total soluble solids to 22 °Brix. To ensure microbial inactivation prior to fermentation, the juice was treated using thermal ultrasonication at 153 W and 50 °C for 11.5 min, and the sterility of the treated juice was confirmed using the standard plate count method [21]. The inocula of *Saccharomyces cerevisiae* (*S. cerevisiae*) and *Lactobacillus plantarum* (*L. plantarum*) were prepared as follows. *S. cerevisiae* was cultivated in the YPD liquid medium [22] at 29 °C for 24 h with shaking, while *L. plantarum* was activated in the MRS liquid medium [23] at 37 °C for 48 h under the same shaking conditions. The cultures were adjusted to an optical density of 2.0 and 1.0 at 600 nm (OD_600_) for *S. cerevisiae* and *L. plantarum*, respectively, corresponding to approximately 1 × 10^8^ CFU/mL. The fermentation was carried out under the optimized conditions determined from preliminary single-factor and orthogonal experiments, with an inoculum ratio of (*S. cerevisiae*: *L. plantarum*) = 1:1 (*v*/*v*), an inoculum amount of 1% (*v*/*v*), a fermentation temperature of 32 °C, and a fermentation time of 36 h; these optimized parameters were used for all subsequent sample preparations unless otherwise stated. For fermentation, the sterilized mulberry juice was aseptically inoculated with *S. cerevisiae*, *L. plantarum*, or a composite inoculum, yielding three fermented beverages: *S. cerevisiae*–fermented mulberry beverage (SM), *L. plantarum*–fermented mulberry beverage (LM), and composite-strain–fermented mulberry beverage (CM). Unfermented mulberry juice (MJ) served as the control. After fermentation, the cultures were centrifuged at 8000 r/min for 10 min at 4 °C, and the resulting supernatants were collected for physicochemical analyses. All experiments were performed in triplicate.

#### 2.2.1. One-Way ANOVA and Orthogonal Experimental Design on Mulberry Fermentation

To assess the influence of major fermentation variables on the characteristics of mulberry fermentation, single-factor experiments were first carried out with inoculum ratio (*Saccharomyces cerevisiae*: *Lactobacillus plantarum)*, inoculum amount, fermentation temperature, and fermentation time as independent factors. The response indicators included sugar content, total sugar, pH, and total acidity of the fermentation broth. In a preliminary comparison, ethanol generation was also monitored under selected fermentation conditions (inoculum ratio 1:1, inoculum amount 1% *v*/*v*, 36 h, 32 °C). The *S. cerevisiae*-fermented sample produced 6.2% (*v*/*v*) ethanol, whereas no detectable ethanol was observed in the composite-strain fermentation. This is attributed to the fact that *L. plantarum* can secrete end-products such as lactic acid and acetic acid, inhibiting *S. cerevisiae* from producing alcohol [24,25].

In subsequent single-factor experiments, the fixed parameters were set as an inoculum ratio of 1:1 (*v*/*v*), an inoculum amount of 1% (*v*/*v*), a fermentation time of 36 h, and a temperature of 32 °C. The tested ranges were as follows: inoculum ratio, 3:1, 2:1, 1:1, 1:2, and 1:3; inoculum amount, 0.5%, 1.0%, 1.5%, 2.0%, and 2.5%; fermentation time, 12 h, 24 h, 36 h, 48 h, and 60 h; and fermentation temperature, 22 °C, 27 °C, 32 °C, 37 °C, and 42 °C. The impact of each factor on fermentation performance was evaluated by one-way ANOVA.

Based on these results, an orthogonal experimental design (L9 (3^4^)) was adopted to determine the optimal combination of fermentation conditions. The orthogonal experimental design and range analysis (K and R values) were conducted to evaluate the influence of each factor and determine the optimal level combination. K represents the mean response value for each factor level, while R denotes the range. Four factors at three levels were included in the design, and sugar content, pH, and sensory evaluation scores were used as integrated indicators to assess the overall fermentation outcome. The detailed factor levels used in the orthogonal design are listed in Table 1.

#### 2.2.2. Sensory Evaluation Method

Sensory evaluation [26] was carried out to compare the sensory performance of the nine orthogonal fermentation combinations of CM described in Section 2.2.1. A panel of ten trained judges (five males and five females) was recruited, all of whom were well-trained according to ISO guidelines (ISO 8586: 2012) [27] and familiar with the sensory characteristics of fermented beverages. Each 50 mL sample was presented in colorless glass cups under uniform white lighting at 25 °C, and all evaluations were performed under randomized and blind conditions. Panelists evaluated appearance, aroma, and taste according to the scoring criteria listed in Table 2. The maximum scores were 23 points for appearance, 33 points for aroma, and 44 points for taste, based on the weighted subitems shown in the table. To minimize fatigue and carry-over effects, samples were served in randomized order, and judges rinsed their mouths with warm water and rested for at least 30 s between evaluations. A structured scoring system was used to distinguish sensory differences among the nine fermentation treatments.

### 2.3. Chemical Analysis

#### 2.3.1. Basic Physical and Chemical Indicators

The physicochemical properties of the fermented mulberry beverages, including sugar content, pH, total sugars, and total acid, were analyzed using standard analytical methods. Sugar content was determined by placing one to two drops of the sample on a handheld refractometer (Shimadzu, Kyoto, Japan), and the results were expressed in °Brix. The pH was measured directly using a pH meter (PHS-3C, Shanghai INESA Scientific Instrument, Shanghai, China).

Total sugars were determined by the phenol–sulfuric acid colorimetric method [28]: 1 mL of the 100-fold diluted sample was mixed with 1 mL of 5% phenol solution, followed by the addition of 5 mL of concentrated sulfuric acid. After thorough mixing, the reaction mixture was maintained at 25 °C for 30 min, and absorbance was measured at 490 nm using a UV–Vis spectrophotometer (Shimadzu, Shanghai, China). Distilled water served as the blank. Total acid was determined by potentiometric titration with 0.1 mol/L NaOH using a pH meter for endpoint detection. The titration volumes of the sample (V_1_) and blank (V_2_) were recorded, and total acidity was calculated according to the following Equation (1):

(1) $$\mathrm{Total}\;\mathrm{acid}\;(g/L)\;=\frac{\left[c\;\times \;\left(V_{1}-V_{2}\right)\right]\;\times \;0.67\;\times \;1000}{V_{s}}$$

where c is the concentration (mol/L) of the NaOH standard solution, V_1_ and V_2_ are the titration volumes (mL) of the sample and blank, respectively, V_s_ is the sample volume (mL), and 0.67 is the conversion factor for malic acid.

#### 2.3.2. Total Phenolics

Total phenolic content was analyzed following a modified Folin–Ciocalteu method described by Chaudhuri et al. [29]. Briefly, 1 mL of the sample(100-fold) was combined with 5 mL of Folin–Ciocalteu reagent and 4 mL of 7.5% Na_2_CO_3_ solution, and the mixture was kept at 40 °C for 1 h to allow color development. The absorbance was then measured at 765 nm using a UV–Vis spectrophotometer. A calibration curve was established with gallic acid solutions ranging from 0 to 0.10 mg/mL, and the total phenolic content was calculated as gallic acid equivalents (g GAE/L) from the standard curve.

#### 2.3.3. Total Flavonoids

The total flavonoid content was quantified using a modified colorimetric method based on Wairata et al. [30]. A 1 mL aliquot of the 20-fold diluted sample was mixed with 1 mL of 12% (*w*/*v*) AlCl_3_ solution and incubated at 25 °C for 1 h. Absorbance was recorded at 415 nm using a UV–Vis spectrophotometer. Quercetin methanol standards were used to construct the calibration curve, and the total flavonoid concentration of each sample was calculated accordingly.

#### 2.3.4. Total Anthocyanins

The anthocyanin content was determined using the pH differential method [31]. Briefly, 1 mL of the 100-fold diluted sample was mixed with 9 mL of 0.025 M KCl buffer (pH 1.0) and 0.2 M sodium acetate buffer (pH 4.5). Absorbance was measured at 510 and 700 nm using a UV–Vis spectrophotometer. The difference in absorbance (A) between the two pH conditions was calculated using the following Equation (2):

(2) $$A\;=\;{{(A}_{510}-A_{700})}_{\mathrm{pH}\;1}-{{(A}_{510}-A_{700})}_{\mathrm{pH}\;4.5}$$

#### 2.3.5. Superoxide Dismutase (SOD) Activity

Total SOD activity was determined using a commercial assay kit (Beijing Biobank Biotechnology Co., Ltd., Beijing, China) (NBT method, according to the manufacturer’s instructions), following the procedure described by Chen et al. [32]. The measurements were conducted in accordance with the instructions outlined in the manual.

#### 2.3.6. Antioxidant Properties

##### DPPH Radical Scavenging Capacity

The DPPH radical scavenging capacity was evaluated according to the method of Guo et al. [33], with minor modifications. A 0.04 mg/mL DPPH solution was prepared in anhydrous ethanol. For the assay, 2 mL of DPPH solution was mixed with 2 mL of the sample (A_1_), while 2 mL of ethanol was mixed with 2 mL of the sample as the sample blank (A_2_). A mixture of 2 mL DPPH solution and 2 mL ethanol was used as the control (A_0_). All mixtures were incubated at room temperature in the dark for 30 min, and the absorbance was measured at 519 nm using a UV–Vis spectrophotometer. The DPPH radical scavenging rate was calculated using the following Equation (3):

(3) $$\mathrm{DPPH}\;\mathrm{radical}\;\mathrm{scavenging}\;\mathrm{rate}\;=\left(1-\frac{A_{1}-A_{2}}{A_{0}}\right)\;\times \;100\%$$

##### Hydroxyl Radical Scavenging Capacity

The hydroxyl radical scavenging capacity was determined following the method of Li et al. [34], with minor modifications. Fresh FeSO_4_ (9 mM), salicylic acid ethanol (9 mM), and H_2_O_2_ (8.8 mM) solutions were prepared prior to use. A reaction mixture was prepared by combining 1 mL of sample with 1 mL of FeSO_4_ solution and 1 mL of salicylic acid solution, followed by the addition of 1 mL of H_2_O_2_ to initiate the reaction. Reaction mixtures were maintained at 37 °C for 30 min, and absorbance was recorded at 510 nm using a UV–Vis spectrophotometer. Deionized water served as the blank. The scavenging rate was calculated using the following Equation (4):

(4) $$\mathrm{Hydroxyl}\;\mathrm{radical}\;\mathrm{scavenging}\;\mathrm{rate}\;=\left(1-\frac{A_{1}-A_{2}}{A_{0}}\right)\;\times \;100\%$$

where A_0_ denotes the control (1 mL FeSO_4_ + 1 mL salicylic acid ethanol + 1 mL H_2_O_2_), A_1_ represents the sample (1 mL sample + 1 mL FeSO_4_ + 1 mL salicylic acid ethanol + 1 mL H_2_O_2_), and A_2_ corresponds to the reaction mixture without H_2_O_2_ (1 mL sample + 1 mL FeSO_4_ + 1 mL salicylic acid ethanol).

##### ABTS Radical Scavenging Capacity

The ABTS radical scavenging capacity was assessed following the method of Rumpf et al. [35], with minor modifications. ABTS (7 mM) and potassium persulfate (2.45 mM) solutions were freshly prepared and mixed in equal volumes, followed by incubation in the dark at 4 °C for 12 h to generate the ABTS·^+^ radical cation. The resulting ABTS·^+^ stock solution was diluted with phosphate-buffered saline (PBS, pH 7.4) to obtain a working solution with an absorbance of 0.70 ± 0.02 at 734 nm. In the scavenging assay, 1 mL of the 40-fold diluted sample was combined with the ABTS working solution, and the reaction was allowed to proceed for 6 min in the dark at 25 °C. The absorbance was then recorded at 734 nm. The ABTS radical scavenging rate was calculated using the following Equation (5):

(5) $$\mathrm{ABTS}\;\mathrm{radical}\;\mathrm{scavenging}\;\mathrm{rate}\;=\left(1-\frac{A_{1}-A_{2}}{A_{0}}\right)\;\times \;100\%$$

where A_0_ is the absorbance of the ABTS–PBS mixture, A_1_ is that of the sample–ABTS mixture, and A_2_ is that of the sample–PBS mixture.

#### 2.3.7. Volatile Components

The analysis of volatile compounds in mulberry beverages was conducted using HS-SPME-GC-MS, following the method developed by Xie et al. [36]. Volatile compounds in the mulberry beverages were analyzed using headspace solid-phase microextraction coupled with gas chromatography–mass spectrometry (HS-SPME–GC–MS, Shimadzu, Kyoto, Japan). The system operated in full-scan mode under electron impact ionization (70 eV), with the ion source and quadrupole temperatures maintained at 230 °C and 150 °C, respectively, and a detector voltage of 350 V. Volatile constituents were separated under optimized chromatographic conditions, and identification was based on spectral matching with the NIST 17 library, retaining compounds with similarity scores greater than 90%.

#### 2.3.8. Data Statistics and Analysis

All measurements were performed in triplicate. Data processing, statistical analysis, and figure preparation were conducted using OriginPro 2025 (Education Edition; OriginLab Corporation, Northampton, MA, USA). Results are expressed as mean ± standard deviation. Differences among means were evaluated using one-way ANOVA followed by the least significant difference test, with statistical significance defined at *p* < 0.05.

## 3. Results and Discussion

### 3.1. Effects of Different Single Factors on the Sugar Content and pH of Fermented Mulberry Beverages

**Figure 1:**
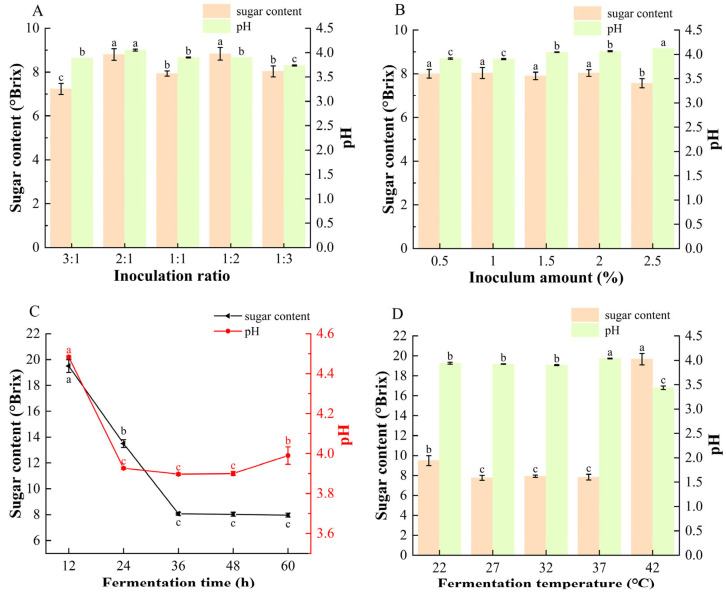
Effects of different (**A**) inoculation ratios of *S. cerevisiae* to *L. plantarum* (inoculum amount fixed at 1%, 36 h, 32 °C), (**B**) inoculation amounts (ratio fixed at 1:1, 36 h, 32 °C), (**C**) fermentation time (inoculum ratio 1:1, inoculum amount 1%, 32 °C), and (**D**) fermentation temperature (inoculum ratio 1:1, inoculum amount 1%, 36 h) on the sugar content and pH of composite-strain fermented mulberry beverage. Data are presented as mean ± standard deviation (*n* = 3). Different letters indicate significant differences between different beverages (ANOVA, *p* < 0.05).

As shown in Figure 1B,C, competition for carbon sources between *S. cerevisiae* and *L. plantarum* leads to distinct sugar-utilization and acid-production behaviors during fermentation [37,38]. As the inoculum ratio changed, a higher proportion of *S. cerevisiae* (3:1) resulted in more rapid sugar consumption, whereas a higher proportion of *L. plantarum* (1:3) produced a more acidic beverage, consistent with the known metabolic characteristics of the two strains [18]. Across all inoculum levels, the final sugar content stabilized at approximately 8 °Brix, except at 2.5% inoculum, where the lowest value of 7.5 °Brix was observed. The pH of the fermented mulberry beverages increased from 3.90 to 4.12 as the inoculum amount increased. This trend may be attributed to the malolactic activity of *L. plantarum*, which converts malic acid to lactic acid during fermentation. Previous studies reported that lactic acid fermentation reduced malic acid content in hawthorn by approximately 90%, supporting this interpretation [39]. Excessive inoculation levels may also accelerate substrate depletion and metabolic rates, thereby limiting the accumulation of bioactive compounds [37].

As shown in Figure 1C,D sugar levels decreased rapidly within the first 36 h before stabilizing, indicating fast initial substrate consumption followed by entry into the stationary phase. The decline in pH during this period reflects the microbial conversion of sugars into organic acids. A similar trend was confirmed under optimized fermentation conditions (Figure S1). Fermentation temperature also strongly affected microbial metabolism. At 42 °C, high residual sugar together with low pH suggested inhibited sugar utilization at elevated temperature, while *L. plantarum* remained active in acid production.

### 3.2. Orthogonal Optimization of Fermentation Conditions

Table 3 summarizes the orthogonal optimization results for the fermentation process. The optimal process identified was A_2_B_2_C_3_D_2_ when sugar content was the primary criterion, whereas A_1_B_2_C_3_D_2_ was optimal when considering pH and sensory quality. Given the importance of sensory attributes in product acceptability, the final optimal fermentation conditions were established as A_1_B_2_C_3_D_2_, corresponding to an inoculation ratio of 2:1, an inoculation amount of 1%, a fermentation time of 60 h, and a fermentation temperature of 32 °C. Under these optimized conditions, the fermented mulberry beverage exhibited a sugar content of 7.5 ± 0.2 °Brix, a pH of 4.12 ± 0.12, and a sensory evaluation score of 89 ± 1.34.

### 3.3. Analysis of the Physicochemical Properties of the Mulberry Beverages During the Fermentation Process

**Figure 2:**
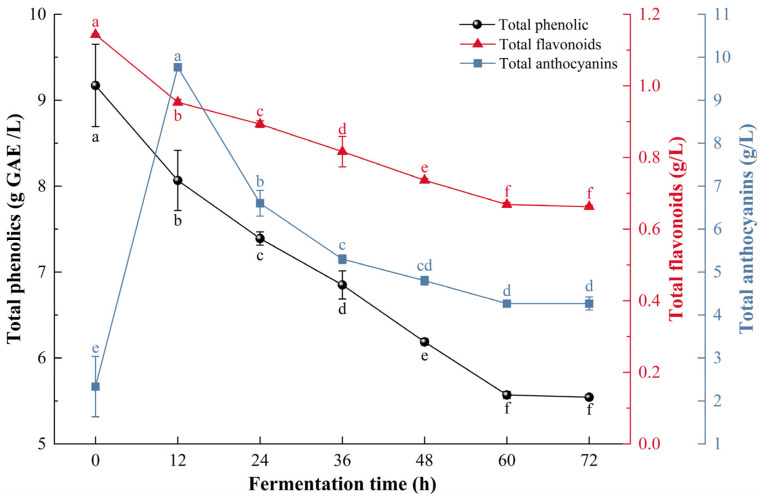
Changes in total phenolics, total flavonoids and total anthocyanins during the fermentation process of composite-strain fermented mulberry beverage. Data are presented as mean ± standard deviation (*n* = 3). Different letters indicate significant differences (*p* < 0.05).

As shown in Figure 2, total phenolic decreased from 9.17 ± 0.48 g/L to 5.54 ± 0.03 g/L during fermentation, while total flavonoids decreased from 1.14 ± 0.01 g/L to 0.66 ± 0.01 g/L. These decreases were caused by the combined activity of *S. cerevisiae* and *L. plantarum*. The cell walls of both microorganisms, which contain β-glucan, chitin, and peptidoglycan [40], have strong affinities for polyphenols and flavonoids through hydrogen bonding, hydrophobic interactions, and ionic forces, leading to adsorption losses [41,42]. In addition, microbial metabolism facilitates the hydrolysis of high-molecular-weight polyphenols into smaller free forms [43] and promotes oxidative degradation of phenolic compounds during fermentation [44].

However, the trend of anthocyanin changes is different. During the first 12 h of fermentation, the total anthocyanin content increased from 2.33 ± 0.7 g/L to 9.77 ± 0.06 g/L. followed by a decline that stabilized at 4.27 ± 0.06 g/L by 60 h. Despite this decrease, the final total anthocyanin content in CM exhibited an increase. This trend is likely related to hydrolytic enzymes produced by *L. plantarum* and *S. cerevisiae*, which convert complex phenolic substances or glycosides into simpler, more extractable molecules [45].

### 3.4. Analysis of Phenolic Compounds and Antioxidant Properties of Mulberry Beverages Fermented by Different Bacterial Strains

As shown in Table 4, beverages fermented with *L. plantarum* displayed the highest total sugar and °Brix values. This may result from the release of mannoproteins and glucans from microbial cell walls during fermentation, as well as the possible production of extracellular polysaccharides by *L. plantarum,* both of which can increase the measurable soluble-sugar fraction [20,46]. All fermented beverages exhibited substantially higher total acid levels than MJ, reflecting organic-acid formation by both *S. cerevisiae* and *L. plantarum* [7].

**Figure 3:**
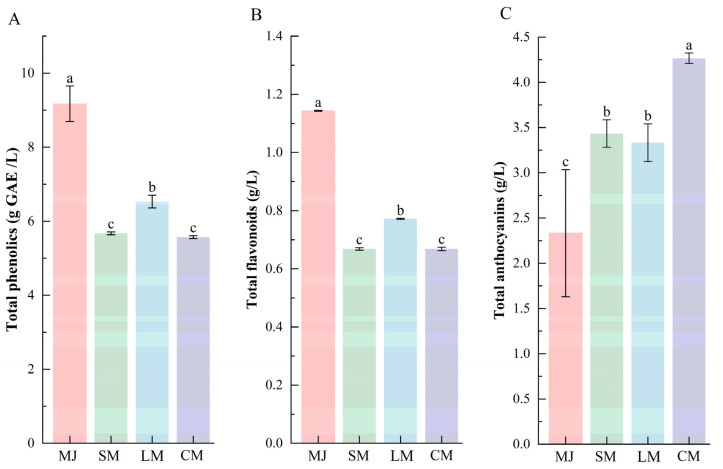
Comparison of total phenolics (**A**), total flavonoids (**B**), and total anthocyanins (**C**) in different types of fermented beverages. Data are presented as mean ± standard deviation (*n* = 3). Different letters indicate significant differences between (*p* < 0.05).

As shown in Figure 3, compared to mulberry juice, the total phenolic and flavonoid contents decreased in all three fermented beverages (SM, LM, and CM). These decreases can be attributed not only to microbial adsorption but also to the biotransformation of polyphenols by *L. plantarum* and *S. cerevisiae* into smaller, more bioactive molecules. Such low-molecular-weight products exhibit higher reactivity, leading to further reactions with other substances [25,47]. Additionally, compounds such as pyruvate and acetaldehyde, produced by *S. cerevisiae* fermentation, can form macromolecular derivatives by interacting with phenolic substances. This process also contributes to the reduction in total phenolic and flavonoid content [44].

In contrast, the total anthocyanin content increased across all fermented beverages, with SM and LM showing comparable levels, whereas CM displayed the highest accumulation (Figure 3C). This enhancement can be attributed to several synergistic mechanisms during fermentation. First, hydrolytic enzymes released by *S. cerevisiae*, including cellulase and pectinase, disrupt plant cell walls and liberate bound anthocyanins, while carbon dioxide generated during yeast metabolism creates a mildly anaerobic environment that helps mitigate oxidative degradation of anthocyanins [48]. Additionally, *L. plantarum* secretes β-glucosidase, which hydrolyzes anthocyanin glycosides into more soluble aglycones [49], and its deglycosylation toward phenolic compounds releases additional soluble phenolic substances [50]. Moreover, the composite-strain fermentation system stabilized the beverage pH within a range of 4.0–4.4, a condition more favorable for anthocyanin stability compared to single-strain fermentations, which are prone to pH fluctuations or excessive acidification [48,50].

**Figure 4:**
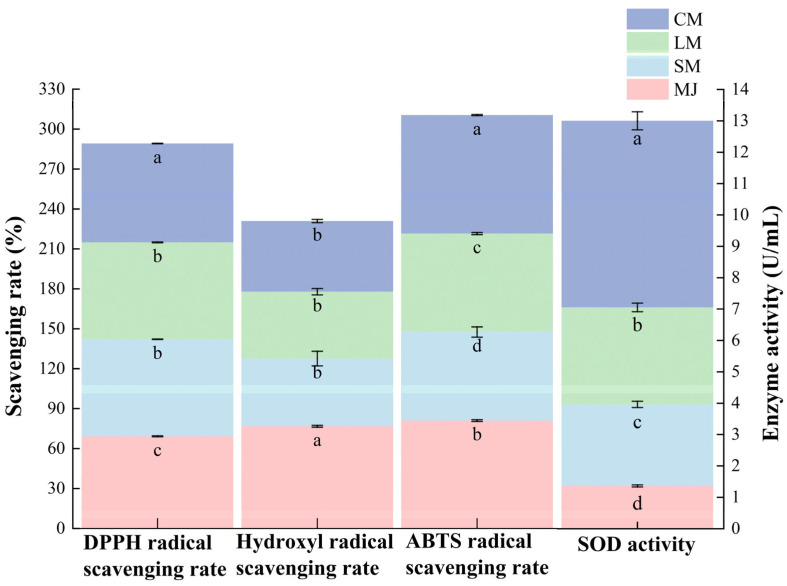
DPPH radical scavenging rate, hydroxyl radical scavenging rate, ABTS radical scavenging rate and SOD activity of different types of mulberry beverages. (DPPH radical scavenging rate, hydroxyl radical scavenging rate, and ABTS radical scavenging rate are plotted on the left vertical axis, while SOD enzyme activity is plotted on the right vertical axis). Data are presented as mean ± standard deviation (*n* = 3). Different letters indicate significant differences (*p* < 0.05).

As shown in Figure 4, the CM exhibited the highest DPPH radical scavenging capacity, achieving a scavenging rate of 74.3%. This is followed by the SM and LM, which exhibit scavenging rates of 72.9 ± 0.2% and 72.77 ± 0.25%. The MJ displays the lowest scavenging rate at 69.23 ± 0.35%. These findings suggest that fermentation significantly enhances DPPH radical scavenging activity. The antioxidant activity of phenolic compounds depends on their chemical structure and is influenced by the groups attached to their phenolic base [51,52]. During this process, the lone pair of electrons on the nitrogen atom of DPPH is reduced to the corresponding hydrazine by accepting a hydrogen atom from antioxidants. The observed in DPPH radical scavenging rate may be attributed to fermentation promoting the presence of compounds with proton-donating properties [50].

**Figure 5:**
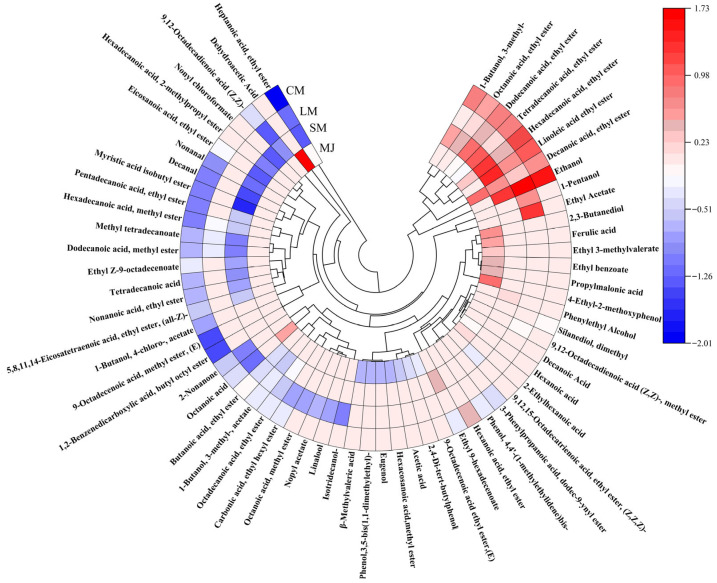
Circular heat map of volatile components in different mulberry beverages. (Perform normalization on the data by taking the logarithm of the original values, then use the resulting numbers to generate a circular heatmap).

As shown in Figure 5, MJ exhibited the highest hydroxyl radical scavenging capacity at 76.76 ± 0.70%. The hydroxyl radical scavenging capacity of fermented mulberry beverages were lower, recorded at 50.84 ± 5.51%, 50.24 ± 2.39%, and 53.09 ± 1.24%. The observed decrease in hydroxyl radical scavenging capacity following fermentation may be linked to a decline in total phenolic and total flavonoid content. Additionally, microbial fermentation may deglycosylate or degrade small-molecule phenolic compounds. These transformation processes could modify the quantity and positioning of phenolic hydroxyl groups, consequently diminishing the reaction rate and efficiency of these compounds in their interaction with hydroxyl radicals [28].

Fermentation significantly altered the ABTS radical scavenging capacity of mulberry beverages. Compared to MJ, SM and LM exhibited reduced ABTS radical scavenging capacity, while CM showed markedly higher ABTS radical scavenging rates than SM and LM. The association between phenolic compounds—particularly flavonoids—and antioxidant activity may relate to acidity and the chemical structure of phenolic compounds [53]. This relationship may also involve resonance between free electron pairs on phenoxy and benzene rings, which enhances electron delocalization and imparting partial negative charge, thereby improving their antioxidant activity against free radicals [54].

A similar trend was observed for SOD activity, which was substantially higher in CM than in the other beverages. SOD is an intracellular antioxidant enzyme widely present in *S. cerevisiae* and *L. plantarum*, and its activity increases with microbial proliferation and metabolic intensity [55]. They catalyze the dismutation of superoxide anion radicals into hydrogen peroxide and oxygen, thus reducing the damage induced by oxidative stress [32].

### 3.5. Analysis of Volatile Components

HS-SPME-GC-MS is a detection technique utilized for the separation and identification of complex components, providing advantages such as safety, accuracy, and high separation capability [56]. Through HS-SPME-GC-MS analysis, a total of 27 aroma compounds were identified in MJ. comprising 1 alcohol, 9 acids, 10 esters, and 4 phenols, and other compounds such as naphthalenes and hydrocarbons. 31 aroma compounds were detected in SM, including 3 alcohols, 24 esters, 2 acids, and 2 aldehydes. LM exhibited 26 aroma compounds, which included 4 alcohols, 19 esters, 2 acids, and 1 ketone. In CM, a total of 37 aroma compounds were detected, consisting 4 alcohols, 26 ester, 3 acid, 2 aldehyde, 1 ketone, and 1 phenol. Thus, composite fermentation significantly enhanced the diversity of volatile compounds in mulberry beverages, resulting in richer aroma profiles compared to single-strain fermentation. The interaction between *L. plantarum* and *S. cerevisiae* facilitated the production of diverse volatile compounds, consistent with findings reported by Zhang et al. [37,57].

Figure 5 Circular heat map of volatile components detected in mulberry beverages (MJ, SM, LM, and CM). Colors represent the normalized relative abundance of each compound, with red indicating higher abundance and blue indicating lower abundance. Compounds are grouped according to chemical classes, including esters, alcohols, acids, aldehydes, ketones, and phenolic derivatives.

Alcoholic compounds represent a significant category of flavor constituents in fermented beverages. In CM, three additional alcohols are detected compared to MJ, primarily resulting from the production of alcohol through sugar metabolism by *S. cerevisiae* and *L. plantarum*. *S. cerevisiae* fermentation generates alcohols via two pathways: the previously mentioned sugar metabolism, and the enzyme-catalyzed conversion of amino acids into keto acids, followed by aldehyde formation and subsequent alcohol synthesis. Ethanol is produced through sugar metabolism by microbial strains during fermentation, while other alcohols arise from enzyme-catalyzed amino acid reactions [58]. Furthermore, alcoholic compounds are originate from metabolic byproducts generated by *L. plantarum* and *S. cerevisiae* through processes such as amino acid decarboxylation, dehydrogenation, or lipid metabolism during fermentation [59]. Amyl alcohol imparts whiskey and banana notes, phenethyl alcohol contributes a sweet, rose-like floral aroma, enhancing the freshness and fruitiness of fermented mulberry beverages. High total alcohol content yields pronounced alcoholic and pungent flavors, leading to coarse beverage profiles, whereas lower concentrations enhance flavor complexity [25].

Compared to MJ, CM contains 16 additional esters. Esters constitute the most diverse class of volatile compounds in CM and play a vital role in enhancing fruit flavors. *S. cerevisiae* produces esters during fermentation, and ester exchange or hydrolase activity likely contributes to their formation [60]. Additionally, *S. cerevisiae* can ferment sugars into lactic acid and salts, thereby accelerating the production of ester flavor compounds [3]. *L. plantarum* fermentation converts sugars, primarily glucose and fructose, into organic acids and aromatic components, which contribute to the flavor development of fermented beverages [19]. Furthermore, condensation reactions between organic acids and alcohols produced by *L. plantarum* can also form esters [61]. Fermented mulberry beverages exhibit elevated levels of lipid compounds such as ethyl palmitate and ethyl caprylate. Esters impart positive fruity characteristics—for instance, ethyl acetate imparts rose and honey aromas with raspberry-like flavor notes [62]. Esters possess lower odor thresholds than alcohols, exerting a more pronounced influence on the aromatic profile of mulberry beverages. Consequently, low concentrations of esters including butylhexyl phthalate, methyl oleate, and ethyl heptanoate may also positively influence the flavor profile of mulberry beverages [63].

Fermented beverages contain 3 to 7 fewer types of acids than MJ. This reduction may result from esterification reactions between acids and alcohols during fermentation, or from *S. cerevisiae* converting organic acids into alcohols in the later stages of fermentation [64]. Moderate acidity enhances the fresh fruit flavor of beverages, harmonizes overall palatability, inhibits the hydrolysis of corresponding aromatic esters, and acts as a precursor for synthetic ester flavor compounds. At levels near the odor threshold, it contributes complexity to the flavors of fermented beverages [65]. Notably, caproic acid exhibits antimicrobial properties and serves as an important dietary supplement [66].

Fermented mulberry beverages contain relatively low levels of aldehydes and ketones, likely due to their reduction or oxidation into alcohols or acids during the metabolic process of *L. Plantarum* [67]. Research indicated that aldehydes and ketones contribute to fruity flavors at lower concentrations, whereas elevated levels adversely affect the aroma of fruit juices [68].

### 3.6. Principal Component Analysis

**Figure 6:**
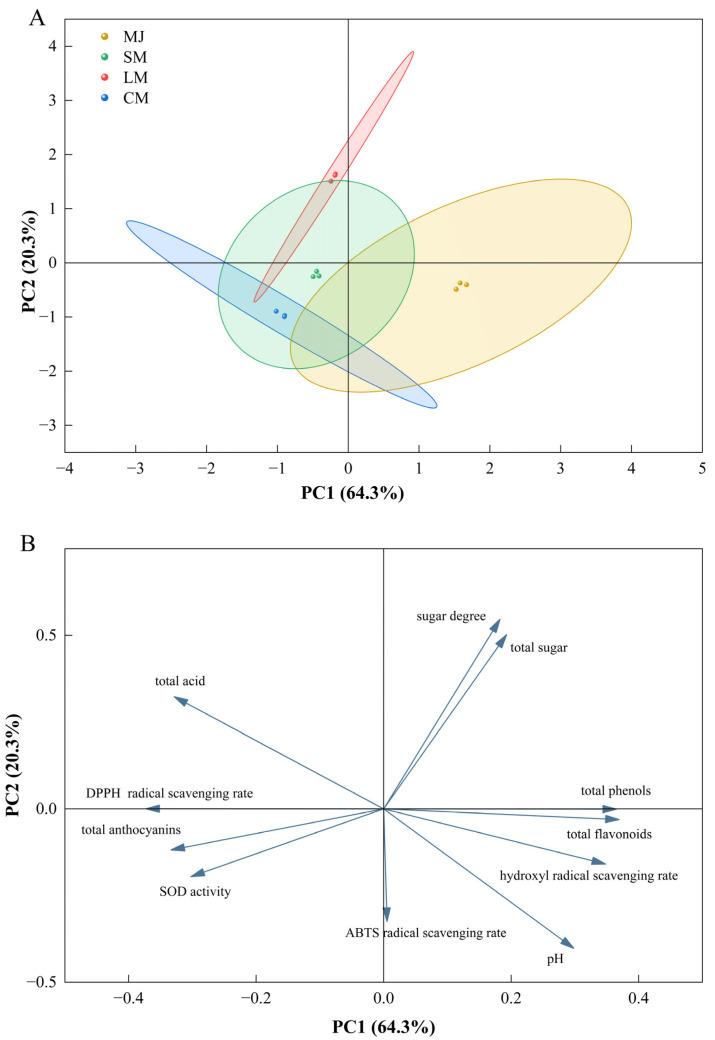
Score plot (**A**) and loading plot (**B**).

Principal component analysis (PCA) was conducted on various indicators of fermented beverages (Figure 6), including total sugars, sugar content, total acids, pH, total phenols, total flavonoids, total anthocyanins, DPPH radical scavenging rate, hydroxyl radical scavenging rate, ABTS radical scavenging rate, and SOD activity. Two principal components, PC1 and PC2, were extracted, accounting for 64.3% and 20.3% of the total variance in the 11-variable system, respectively. The cumulative contribution rate of these two components reached 84.6%. In the score plot, SM, LM, and CM were positioned on the negative side of PC1, while MJ was located on the positive side of PC1.

A loading plot based on eigenvalues (EVs) indicated that EV = 7.07 for PC1 and EV = 2.23 for PC2. Total sugars, sugar content, pH, total phenols, total flavonoids, and the hydroxyl radical scavenging rate were situated in the positive region of PC1, while total acids, total anthocyanins, the DPPH radical scavenging rate, the ABTS radical scavenging rate, and SOD enzyme activity were positioned in the negative region of PC1.

## 4. Conclusions

This study showed that co-fermentation with *S. cerevisiae* and *L. plantarum* can enhance the overall quality of mulberry beverages by strengthening antioxidant activity, enriching the volatile profile, and improving anthocyanin retention compared with single-strain fermentation. These findings suggest that mixed-strain fermentation offers a promising approach for developing mulberry beverages with better functional properties and sensory appeal. However, the dynamic changes in organic acids and phenolic compounds were not monitored in detail, and the work focused on only one specific *S. cerevisiae*–*L. plantarum* pairing under fixed fermentation conditions, which may restrict the broader applicability of the results. Future studies should therefore examine additional strain combinations and ratios, extend validation to other fruit substrates, and incorporate multi-omics analyses to clarify the microbial interactions and biochemical pathways responsible for quality improvements during mixed-strain fermentation.

However, a limitation of this study is the lack of a detailed analysis of the changes in organic acids and phenolic compounds during fermentation. Future work should focus on this aspect to better elucidate the transformation mechanisms of functional components in mulberries by the composite microbial strain. Moreover, because the fermentation responses observed here were obtained under a specific strain combination and controlled processing conditions, the findings may not be directly extendable to other fruit substrates or microbial systems.

## Figures and Tables

**Table 1:** Orthogonal optimization of the fermentation process.

Level	Factors			
	Inoculation Ratio (*v*/*v*) (A)	Inoculum Amount/% (*v*/*v*) (B)	Fermentation Time/h (C)	Fermentation Temperature/°C (D)
−1	2:1	0.5	36	27
0	1:1	1.0	48	32
1	1:2	1.5	60	37

The inoculation ratio refers to the volumetric ratio of *Saccharomyces cerevisiae*: *Lactobacillus plantarum*.

**Table 2:** Sensory evaluation standard.

Item		Score
Appearance	color: dark purple	6
	clarity: shaking in the sunlight is relatively clear	6
	overall appearance: the color is uniform and consistent, with a smooth texture and a lustrous sheen	11
Aroma	fruitiness: it has the distinctive aroma of blackberries	6
	mellowness: rich aroma	6
	acidity: moderately sour	6
	overall aroma: the aroma is harmonious and balanced, with a distinctive mulberry flavor	15
Taste	fruitiness: it has the flavor of mulberries	6
	sweetness, acidity: Sweet and sour just right, with no off-flavors	6
	aftertaste: Sweet aftertaste	6
	texture: uniform and stable texture	6
	overall mouthfeel: delicate texture, harmonious and balanced flavor	20

**Table 3:** Orthogonal optimization results of fermentation process.

Treatment	Inoculation Ratio (A)	Inoculum Amount/% (B)	Fermentation Time/h (C)	Fermentation Temperature/°C (D)	Sugar Content/°Brix	pH	Sensory Evaluation Score
1	2:1	0.50	36.00	27.00	15.00 ± 0.62	3.81 ± 0.31	75 ± 0.31
2	2:1	1.00	48.00	32.00	8.90 ± 0.24	4.05 ± 0.07	89 ± 0.11
3	2:1	1.50	60.00	37.00	7.50 ± 0.08	3.93 ± 0.24	85 ± 0.25
4	1:1	0.50	48.00	37.00	9.00 ± 0.31	3.67 ± 0.41	80 ± 0.29
5	1:1	1.00	60.00	27.00	7.10 ± 0.15	3.84 ± 0.32	85 ± 0.23
6	1:1	1.50	36.00	32.00	10.00 ± 0.11	3.84 ± 0.31	79 ± 0.30
7	1:2	0.50	60.00	32.00	7.20 ± 0.11	3.80 ± 0.37	82 ± 0.26
8	1:2	1.00	36.00	37.00	10.00 ± 0.43	3.78 ± 0.39	77 ± 0.28
9	1:2	1.50	48.00	27.00	8.50 ± 0.21	3.73 ± 0.40	80 ± 0.24
K1	10.47	10.43	11.67	10.20			
K2	8.70	8.67	8.80	8.73			
K3	8.70	8.67	7.30	8.83			
R	1.87	1.76	4.36	1.46			
Optimal sugar content conditions: A_2_B_2_C_3_D_2_							
K1	3.93	3.76	3.81	3.79			
K2	3.79	3.88	3.82	3.90			
K3	3.76	3.84	3.85	3.79			
R	0.17	0.12	0.04	0.12			
Optimal pH conditions: A_1_B_2_C_3_D_2_							
K1	83.00	79.00	77.00	80.00			
K2	81.33	83.67	83.00	83.33			
K3	79.67	81.33	84	80.67			
R	3.33	4.67	7.00	3.33			
Optimal conditions for sensory evaluation: A_1_B_2_C_3_D_2_							

K1, K2, and K3 represent the mean response values of level 1, level 2, and level 3 for each factor (A–D), respectively. R denotes the range (R = Kmax − Kmin), which reflects the relative influence of each factor. Data are presented as mean ± standard deviation (*n* = 3).

**Table 4:** Comparison of the basic properties of four beverages.

Compound	MJ	SM	LM	CM
Total sugar (g/L)	239.72 ± 3.46 ^a^	88.02 ± 6.63 ^d^	204.86 ± 2.45 ^b^	105.36 ± 1.56 ^c^
Sugar content (°Brix)	22 ± 0.10 ^a^	7.8 ± 0.10 ^c^	19 ± 0.25 ^b^	7.5 ± 0.2 ^c^
Total acid (g/L)	1.92 ± 0.12 ^d^	6.17 ± 0.13 ^c^	11.98 ± 0.14 ^a^	9.74 ± 0.09 ^b^
pH	5.34 ± 0.01 ^a^	4.64 ± 0.03 ^b^	3.50 ± 0.02 ^d^	4.12 ± 0.03 ^c^

Data are presented as mean ± standard deviation (*n* = 3). Different lowercase superscript letters within the same raw indicate significant differences (*p* < 0.05).

## Data Availability

The original contributions presented in the study are included in the article/Supplementary Materials. Further inquiries can be directed to the corresponding author.

## Supplementary Materials

The following supporting information can be downloaded at https://www.mdpi.com/article/10.3390/foods14244312/s1, Figure S1: Changes in total sugar and sugar content (A) and total acid and pH (B) of composite-strain fermented mulberry beverage during fermentation. Different letters indicate significant differences (ANOVA, *p* < 0.05).
