# Supplementary material for: Optimization of the Fermentation Process for a Mulberry Beverage Using Composite Microbial Strains and a Study on Its Physicochemical Properties
Source: Foods. 2025 Dec 15;14(24):4312. doi: 10.3390/foods14244312 (PMC12732356; doi:10.3390/foods14244312)
Supplement: Supplementary file 1 [file foods-14-04312-s001.zip › foods-3953611-supplementary.pdf]

Supplementary Materials

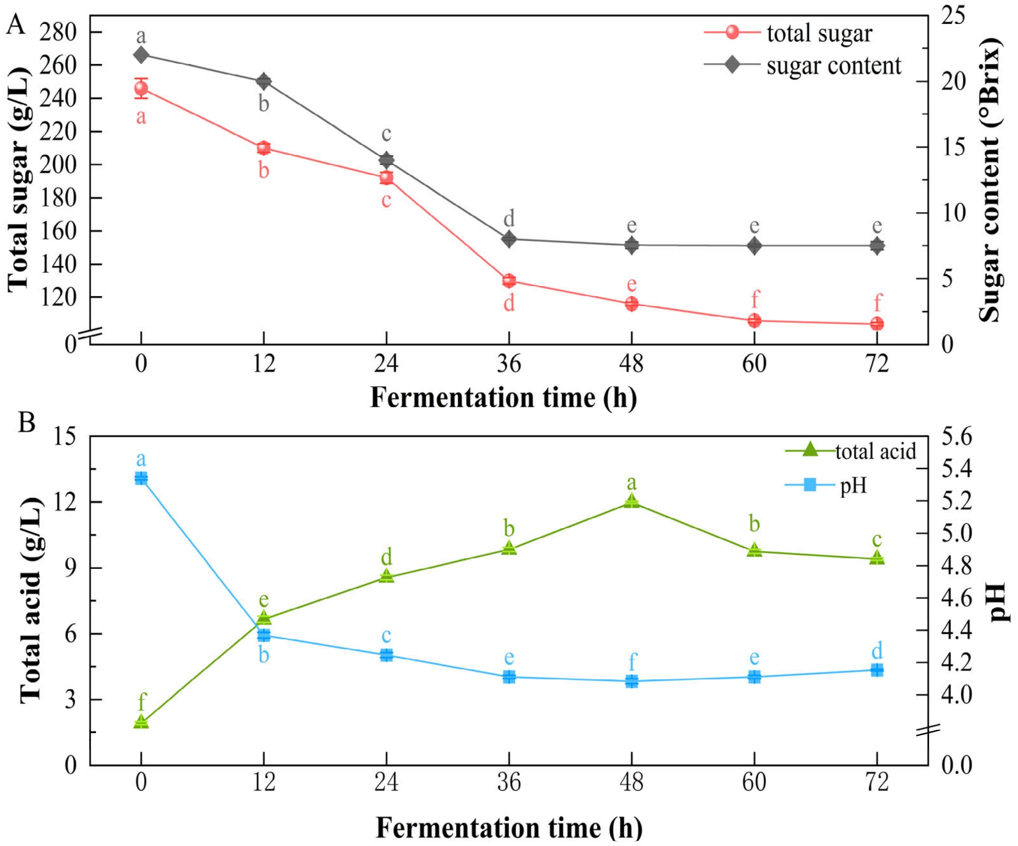

Figure S1: Changes in total sugar and sugar content (A) and total acid and pH (B) of composite-strain fermented mulberry beverage during fermentation. Different letters indicate significant differences (ANOVA, $p < 0.05$).
